# Mature Epitope Density - A strategy for target selection based on immunoinformatics and exported prokaryotic proteins

**DOI:** 10.1186/1471-2164-14-S6-S4

**Published:** 2013-10-25

**Authors:** Anderson R Santos, Vanessa Bastos Pereira, Eudes Barbosa, Jan Baumbach, Josch Pauling, Richard Röttger, Meritxell Zurita Turk, Artur Silva, Anderson Miyoshi, Vasco Azevedo

**Affiliations:** 1 Molecular and Cellular Genetics Laboratory, Universidade Federal de Minas Gerais, Belo Horizonte, Minas Gerais, Brazil; 2 Computational Biology Research Group, Department of Mathematics and Computer Science, University of Southern Denmark, Campusvej, Odense M, Denmark; 3 DNA Polymorphism Laboratory, Universidade Federal do Pará, Campus do Guamá, Belém, Pará, Brazil; 4 Computational Systems Biology group, Max Planck Institute for Informatics, Campus E2.1, Saarbrücken, Germany; 5 Faculty of Computing, Universidade Federal de Uberlândia, Campus Santa Mônica, Uberlândia, Minas Gerais, Brazil

## Abstract

**Background:**

Current immunological bioinformatic approaches focus on the prediction of allele-specific epitopes capable of triggering immunogenic activity. The prediction of major histocompatibility complex (MHC) class I epitopes is well studied, and various software solutions exist for this purpose. However, currently available tools do not account for the concentration of epitope products in the mature protein product and its relation to the reliability of target selection.

**Results:**

We developed a computational strategy based on measuring the epitope's concentration in the mature protein, called Mature Epitope Density (MED). Our method, though simple, is capable of identifying promising vaccine targets. Our online software implementation provides a computationally light and reliable analysis of bacterial exoproteins and their potential for vaccines or diagnosis projects against pathogenic organisms. We evaluated our computational approach by using the *Mycobacterium tuberculosis *(*Mtb*) H37Rv exoproteome as a gold standard model. A literature search was carried out on 60 out of 553 *Mtb*'s predicted exoproteins, looking for previous experimental evidence concerning their possible antigenicity. Half of the 60 proteins were classified as highest scored by the MED statistic, while the other half were classified as lowest scored. Among the lowest scored proteins, ~13% were confirmed as not related to antigenicity or not contributing to the bacterial pathogenicity, and 70% of the highest scored proteins were confirmed as related. There was no experimental evidence of antigenic or pathogenic contributions for three of the highest MED-scored *Mtb *proteins. Hence, these three proteins could represent novel putative vaccine and drug targets for *Mtb*. A web version of MED is publicly available online at http://med.mmci.uni-saarland.de/.

**Conclusions:**

The software presented here offers a practical and accurate method to identify potential vaccine and diagnosis candidates against pathogenic bacteria by "reading" results from well-established reverse vaccinology software in a novel way, considering the epitope's concentration in the mature portion of the protein.

## Background

Tuberculosis (TB) has been one of the major causes of morbidity and mortality worldwide for centuries, and control of the spread of *Mycobacterium tuberculosis *(*Mtb*) infection remains a public health priority [1]. More than 9 million new cases of TB in humans arise every year, resulting in nearly 2 million deaths worldwide [2]. Bacille Calmette-Guérin (BCG), the current vaccine for the treatment of TB, has its limitations; although it is protective against severe childhood TB, it does not satisfactorily prevent the pulmonary disease in adults [3]. Effective prophylactic and therapeutic immunization is a key strategy for global epidemic control [1]. Novel TB vaccine candidates include BCG or recombinant BCG (rBCG) strains, which are used in heterologous prime-boost strategies as a prime vaccination [4]. Booster vaccinations can include viral vectors that express immunodominant *Mtb *antigens or fusion proteins of these antigens, combined with adjuvanticity to ensure immunogenicity [5]. Many *Mtb *antigens, including Ag85A, Ag85B, TB10.4 and ESAT-6, have been tested as vaccine candidates; however, these have not been shown to be successful at treating TB [6]. Consequently, discovering new antigens continues to be a crucial factor for the successful development of vaccines against TB [7].

Exported proteins are currently the main target for Reverse Vaccinology (RV) due to their essential role in host-pathogen interactions [8]. Examples of this interaction include the following: (i) adherence to host cells; (ii) invasion of the cell to which there was compliance; (iii) damage to host tissues; (iv) resistance from the defense machinery of the cells to environmental stress; and (v) mechanisms for subversion of the host's immune response [9,10]. In general, RV reveals a great number of proteins that could constitute potential targets of vaccine candidates that then have to be confirmed via cost-intensive and time-consuming wet-lab experiments. However, incorporating immunoinformatic filters, which identify target proteins with high potential in the RV process, could reduce these drawbacks [11]. Immunoinformatics focuses mainly on small peptides ranging from 8 to 11 residues, called linear epitopes, particularly on those that strongly bind to MHC class I molecules. Just one epitope per protein can be enough to create an immune response in the host [12-14]. Bioinformatic techniques to search for epitopes are well understood and available, but can sometimes lead to high false positive rates [15]. Despite this drawback, epitope predictors are capable of identifying weak or even strong epitope motifs that have been experimentally neglected [16].

Epitope density has been described in research as a function of "hot spots" or regions with enriched MHC class II binding epitopes [16]. This work reported 544, 609 and 757 15mers peptides binding to three, two and just one of the molecules HLA-DR1, -DR2, and -DR4, respectively. An analysis of two of the 61 proteins examined in that study showed that Ag85B and MPT63 contain, respectively, 30 and 23 peptides with highest binding to MHC molecules; however, experimental data was only available for 10 peptides derived from MPT63.

Asking whether specific defined domains have high epitope densities, one study found that signal peptides and trans-membrane domains have exceptionally high epitope densities [17]. This work computed the high epitope density of signal peptides using *in silico *methods which corroborate with the high percentage of identified signal peptide epitopes in the IEDB (immune epitope database). The enhanced immunogenicity of signal peptides was experimentally confirmed using peptides derived from *Mtb *proteins. High antigen-specific response rates and population coverage to signal peptide sequences were found when compared with non-signal peptide antigens derived from the same proteins. The MED (Mature Epitope Density) concept is similar to epitope density [16]. To demonstrate the potential of MED to uncover bacterial targets for RV, we collected a set of experimental evidence from the literature that demonstrates a relationship between high MED scores and promising targets in *M. tuberculosis *(*Mtb*) strain H37Rv.

## Results

### Allele frequency

**Figure 1:**
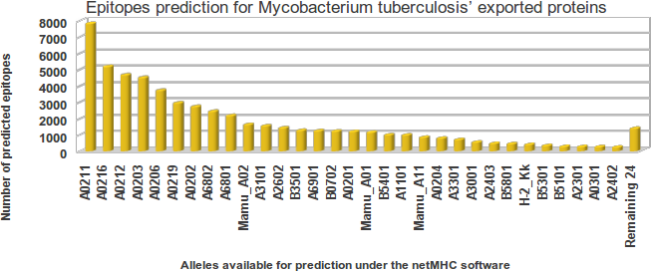
**MHC alleles used to predict MED score**. MHC alleles in the NetMHC software (horizontal axis) and the number of predicted strong binders to epitopes from *Mtb *H37Rv exported proteins (vertical axis).

Figure 1 shows the MHC allele histogram of the predicted epitopes of all 553 *Mtb *H37Rv exported proteins. The horizontal axis represents the alleles available for prediction through the NetMHC software (version 3.0), and the vertical axis represents the absolute number of epitopes predicted by each allele of all exported proteins. The MHC alleles are ordered according to their decreasing number of predicted epitopes. The first five MHC alleles are human and represent 52.32% of all predicted epitopes, the first 15 represent 80.83%, and the last 24 MHC alleles only represent 2.58% of the overall NetMHC epitope prediction.

### Control datasets

**Figure 2:**
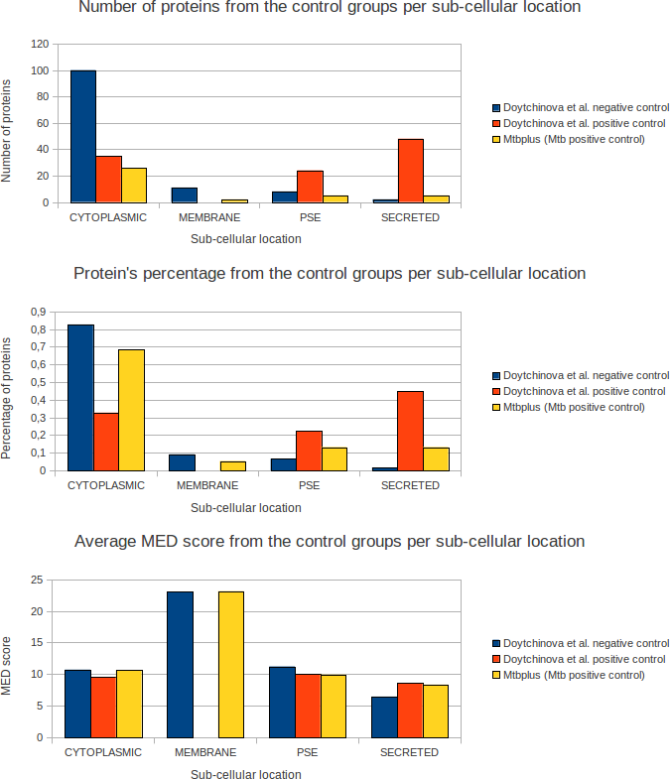
**MED score applied into previous control groups**. Three previously published protein control groups were assessed according to predicted sub-cellular location and the average MED score. Quantities (top panel) and percentages (middle panel) of proteins, plus the average MED scores per predicted local sub-cellular, were analyzed. **These control groups include *M. tuberculosis *antigenic proteins **obtained from the AntigenDB site that were **observed eliciting immune cellular responses and the control groups presented by **Doytchinova *et al*. (2007).

In the Figure 2, the control groups were divided in panels exhibiting protein quantity, percentage regarding this quantity and the average MED score. The horizontal axis of all three panels states the predicted sub-cellular location (cytoplasmic, membrane bound, PSE or secreted) for three groups of proteins: the Doytchinova *et al*. (2007) control groups (positive and negative control groups represented by Dplus and Dminus, respectively) and an *Mtb *positive control group (Mtbplus) taken from the AntigenDB. The vertical axis displays the data (from top to bottom): number of proteins, the percentage represented by the number of proteins and the average MED score for each control group. The number of proteins (top panel) and percentage (middle panel) predicted as cytoplasmic represent the majority for both Dminus and Mtbplus groups, while the Dplus group has more predicted exported proteins. Curiously, the Mtbplus group has the majority of cytoplasmic predicted proteins, which is surprising as it was expected that the majority of antigenic proteins would be exported to the extracellular milieu, as observed in the Dplus group that contains several pathogenic organisms.

Two results should be noted in the bottom panel. Firstly, the average MED scores were very similar among the three control groups, showing that MED is not necessarily a binary statistic classifier for targets but also a continuous statistic measure capable of defining the preferable targets; however, when significant differences between MED scores are shown, it can be used just like a binary classifier. This procedure was assessed in the evidence dataset shown in the next section. Secondly, the average MED score for proteins predicted as membrane-integral were shown to be twice as great as in the other sub-cellular compartments. This result agrees with other work in which signal peptides and trans-membrane domains were found to have exceptionally high CD8+ T cell epitope densities [17].

### Evidence dataset

**Figure 3:**
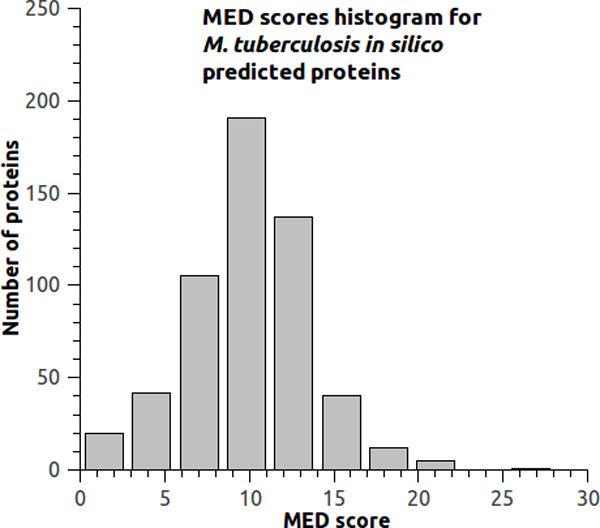
**MED scores from *M. tuberculosis***. MED score histogram for *Mtb *H37Rv exported proteins. Data in Tables 1 and 2 are situated in the extremities of this graph.

Figure 3 shows a histogram representing the distribution of MED scores for all 553 *Mtb *exported proteins. As seen in Table 1, MED scores range from 15.67 to 27.00 nM/mer, with the highest MED score data set represented on the far right side of Figure 3. These values strongly contrast with MED scores of Table 2, which are between 0.00 and 3.19 nM/mer, with the lowest MED score dataset represented on the far left side of Figure 3. As mentioned in the previous section, the MED score is not a binary classifier but is also capable of analyzing proteins scored within these extremely different ranges, allowing us to develop evidence for the general importance of MED scores.

**Table 1:** MED highest-scored proteins.

Genome Locus	N	d	MED (nM/mer)	Local	Evidence	Unique publication identifier
Rv2452c	14	18	27,00	SEC	favorable	10.1046/j.1365-2958.1999.01593.x

Rv1811	66	108	21,34	PSE	favorable	PMID:10760138

Rv3018c	145	234	20,72	PSE	favorable	10.1099/jmm.0.47565-0, 10.1046/j.1365-2958.1999.01593.x, 10.1016/j.vaccine.2004.08.046

Rv1489	37	63	20,36	PSE	favorable	10.1186/1471-2180-10-132, 10.1021/pr0500049, 10.1016/j.tube.2008.01.003

Rv0847	58	98	19,89	SEC	favorable	10.1016/j.tube.2006.01.014, 10.1016/j.tube.2006.01.014

Rv0436c	78	123	19,14	PSE	favorable	10.1074/jbc.M004658200

Rv0116c	117	214	17,61	SEC	favorable	10.1099/mic.0.024802-0

Rv1841c	167	308	17,33	PSE	favorable	10.1128/jb.184.4.1112-1120.2002

Rv2339	224	437	17,25	PSE	favorable	10.1093/molbev/msm111

Rv0589	195	364	17,10	PSE	favorable	10.1007/s11010-011-0733-5

Rv1158c	107	189	17,07	SEC	favorable	10.1016/j.tube.2004.09.005

Rv0286	129	242	17,04	PSE	favorable	10.1128/IAI.70.12.6996-7003.2002

Rv3497c	161	314	16,87	SEC	favorable	10.1073/pnas.1631248100

Rv1967	151	305	16,53	SEC	favorable	10.1111/j.1574-695X.2010.00677.x

Rv1620c	156	311	16,52	PSE	favorable	10.1073/pnas.1003219107, 20090285847

Rv3000	86	167	16,04	PSE	favorable	10.1016/j.tube.2006.01.014

Rv2690c	64	126	16,03	PSE	favorable	Patent 7393540

Rv0804	87	175	15,85	SEC	favorable	10.1107/S1744309108031679

Rv0598c	58	104	15,85	SEC	favorable	PMID:12657046

Rv3693	203	404	15,69	SEC	favorable	10.4049/jimmunol.1002212, 10.1002/pmic.200600853

Rv2262c	100	206	15,69	PSE	favorable	PMID:12368431

Table 1 lists 21 of the 30 highest MED scored-proteins from the *Mtb *H37Rv exported proteins. Each protein is accompanied by at least a unique publication identifier, which can be doi, Pubmed id or a patent number. A protein can be cited twice or thrice by different publications; some publications cite several proteins. The first columns show the protein locus tags, followed by the number of predicted epitopes (n) and epitope probability as a function of its proportion in the mature protein (d). The MED score is calculated as n divided by d. Evidence can be favorable or contrary based on publication results and the expectation indicated by the MED score.

**Table 2:** MED lowest-scored proteins.

Genome Locus	N	d	MED (nM/mer)	Local	Evidence	Unique publication identifier
Rv0532	59	555	3,19	SEC	contrary	10.1021/pr1005108

Rv0746	77	741	3,11	SEC	contrary	10.1186/1471-2148-6-95, 10.1016/j.micinf.2006.03.015

Rv1468c	37	328	3,03	SEC	contrary	10.1021/pr1005108

Rv3590c	48	542	2,96	SEC	favorable	10.1016/S1672-0229(08)60039-X

Rv3511	66	678	2,91	SEC	favorable	10.1186/1471-2148-6-95

Rv1100	20	160	2,88	PSE	contrary	10.1099/mic.0.27204-0

Rv3312A	4	64	2,69	SEC	contrary	10.1073/pnas.0602304104

Rv3595c	34	400	2,51	SEC	contrary	10.1186/1471-2148-6-95

Rv1091	60	814	2,40	SEC	contrary	10.1186/1471-2148-6-95

Rv3706c	4	50	2,32	PSE	contrary	10.3389/fmicb.2010.00121

Rv3345c	98	1498	2,05	SEC	favorable	10.1186/1471-2148-6-95, 10.1099/mic.0.26660-0

Rv0559c	4	78	2,05	SEC	contrary	10.1371/journal.pone.0007615

Rv3388	44	690	2,03	SEC	contrary	10.1016/j.tube.2003.12.014

Rv0833	52	689	1,75	PSE	favorable	10.1186/1471-2148-6-95

Rv2487c	28	655	1,15	SEC	contrary	Patent EP2207035

Rv3514	43	1448	0,93	SEC	contrary	10.1111/j.1365-2567.2010.03383.x

Rv3508	40	1860	0,71	SEC	contrary	10.1371/journal.pone.0002375, 10.1002/prot.10586

Rv3655c	0	0	0	PSE	contrary	10.1371/journal.pone.0010474

Table 2 lists 18 of the 30 MED lowest-scored proteins from the *Mtb *H37Rv exported proteins. Each protein is accompanied by at least a unique publication identifier, which can be doi, Pubmed id or a patent number. A protein can be cited twice or thrice by different publications; some publications cite several proteins. The first columns in Tables 1 and 2 show the protein locus tags, followed by the number of predicted epitopes (n) and epitope probability as a function of its proportion in the mature protein (d). The MED score is calculated as n divided by d. Evidence can be favorable or contrary based on publication results and the expectation indicated by the MED score.

### MED score limitations

**Figure 4:**
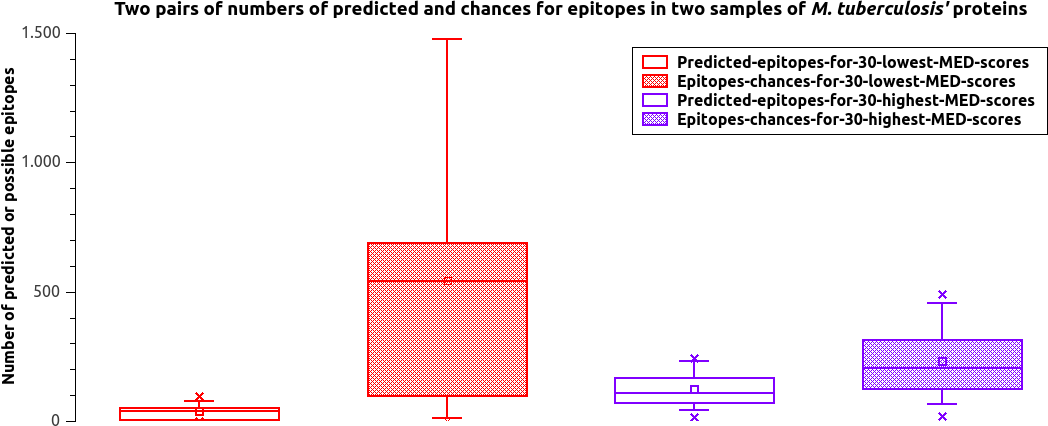
**MED score limitation**. Boxplot of pairs of numerators and denominators within the 60 lowest and highest MED scores from the *Mtb *H37Rv exported proteins. "Predicted" stands for epitope predictions and "Chances" stands for possible 9mers windows in an amino acid sequence's mature portion; both are used in Equation 1. This graph illustrates the major limitation of MED scores: a factor greater than five between the numerator and denominator of a MED score calculation can cover an antigenic protein creating a false negative.

Figure 4 is useful to understand the main limitation of MED scores. It shows two pair of box plots, each pair representing a numerator (predicted epitopes) and a denominator (possibilities or chances for epitopes) that are used in Equation 1. The first pair of boxes show data from the numerator and denominator from the 30 lowest MED scored proteins from the *Mtb *exported proteins, shown at the far left side of Figure 3; the second pair of boxes show data from the 30 highest MED scored proteins from the *Mtb *exported proteins, shown at the far right side of Figure 3. The numerators and denominators were investigated to determine how protein length can influence the MED score. The number of epitopes predicted in the highest-scored subset is more than twice as high as the lowest-scored subset. This result was expected because there is evidence that the highest-scored subset is composed of proteins related to antigenicity or contributing to the bacterial pathogenicity while the majority of the lowest-scored subset is not. The number of possibilities for linear epitopes in the lowest-scored subset is almost three times higher when compared to the highest-scored subset. This numerical difference in the denominators is the major limitation for the MED score strategy, especially for data above the average. Quartiles Q3 and Q4, among those with lowest chances, include half (7/14) the evidence, in contrast to our hypothesis of an existing relation between MED and promising reverse vaccinology targets. These quartiles include denominators between 537 and 1,860 (just one greater than 1,498). Thus, according to the data, MED scores tend to indicate false positives when there is a difference factor of at least five between the number of predictions and the number of epitope possibilities located in the mature amino acid sequence portion. No false positives were observed when this factor was less than two. An interesting result is that the two biggest control groups from Figure 2, Dplus and Dminus, had average factors (fold) of 3.22 and 2.82, respectively.

### MED score sensitivity

**Figure 5:**
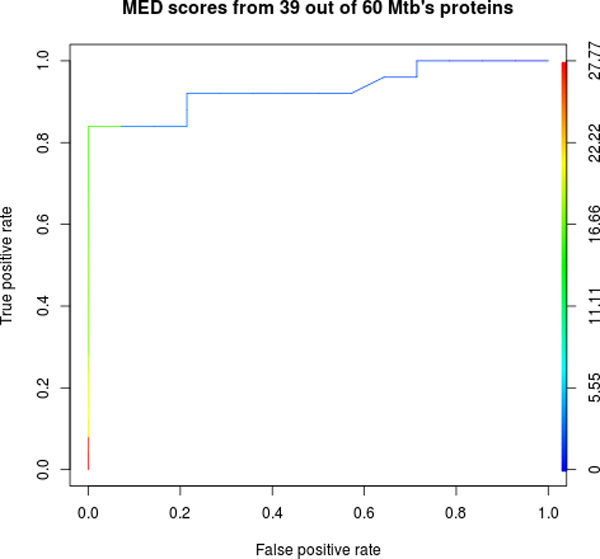
**MED score ROC curve**. Receiver operating characteristic (ROC) curve from the Mature Epitope Density (MED) score calculated for 39 *Mtb *H37Rv exported proteins with favorable or contrary evidence to the MED concept.

Among the 30 proteins that were lowest scored by MED, 14 showed contrary evidence and just four favorable evidence to the MED score concept. Among the 30 highest scored proteins, there was favorable evidence for 21 proteins based on the MED score and no protein with contrary evidence. Among the lowest and highest scored remainders, none showed favorable or contrary evidence related to MED scores. These results were used to create Figure 5 with a ROC curve graph that calculated sensitivities of 84% for MED scores with 7% false positives.

### Novel probable putative *Mtb *antigens

The *Mtb *H37rv proteins Rv0235c, Rv0492A and Rv1004c were predicted to have some of the highest MED scores: 17.78, 20.31 and 18.58 nM/mer, respectively. The former two were predicted to be potentially exposed on the bacterial surface, and the latter was predicted to be secreted. Respectively, there are 78, 43 and 228 predicted epitopes against 138, 73 and 386 epitope chances for these proteins. This is the first published indication of their roles in bacterial antigenicity; MED scoring results suggest these proteins as useful putative targets for future investigations.

## Discussion

### Allele frequency

The available methods for MHC epitope prediction take into account allele frequency in the selection of potential candidates [18,19]. Some alleles are extremely rare; others are specific to some population or widespread [20]. The tools applied here to search for epitopes are not novel, but the way the results are read from standard software tools can be considered a novelty. We proposed to interpret not only epitope prediction from some specific MHC alleles, but from all available alleles. This proposition has a rationale: the idea of assessing the immunogenic potential of a protein, independent of alleles, helps to avoid excluding a protein from a list of *in silico *candidates just because the suitable allele for a specific population was not selected. For example, there are pathogenic organisms that cause different diseases in different hosts, including humans, caprines, ovines, equines, bovines and buffaloes [21-29]. In such cases, it is not reasonable to exclude a single allele from the current limited number available in software tools.

### Control datasets

Even within the Dminus group, the average MED scores were similar to those from the Dplus and Mtbplus groups. Because of this, we focused on predicted exported proteins to create a priority list of targets for the *Mtb *genome, which is a reasonable strategy because one of the main differences between the Dminus and the Dplus groups are the number of predicted cytoplasmic versus exported proteins: 111 and 10 for Dminus versus 35 and 72 for Dplus, respectively. It is more likely that exported proteins interact with the host cells than membrane and cytoplasmic proteins [6,9,10,30]. However, it is important not to neglect proteins that could be exported via non-classical mechanisms. This conclusion can also be drawn out by analyzing the middle panel of Figure 2, where the majority of Mtbplus proteins are classified as cytoplasmic. Medpipe allows the prediction of cytoplasmic targets, but this is the major part of any bacterial genome; medpipe still does not allow differentiating between cytoplasmic proteins without classical exportation motifs and those exported via non-classical pathways.

In addition, it is quite difficult to compare MED scores with previous trained software for antigenic features as such programs tend to be binary classifiers [31-33]. For instance, two control datasets used here were split into training sets (75 proteins) and test sets (25 proteins). Such division does not make sense for MED score because it does not depend on training steps; instead, the MED technique searches for immunological features based on a probable immunological memory concerning epitopes from known pathogens. In this regard, the results obtained with the evidence dataset is more informative because they represent experimental evidence of predictive strengthens or weaknesses of the method.

### Evidence dataset

An extensive literature search for proteins from the well-studied *Mtb *organism gave experimental indication to validate our hypothesis that promising proteins for reverse vaccinology can be revealed based on the overall set of predicted epitopes. When searching for literature evidence, regarding the proteins within the evidence dataset, experimental results of other proteins were also found but not included in this work. This approach was chosen because it is not possible to determine a mean value for MED scores in order to use it as a binary classifier because the number of epitopes predicted per protein can vary significantly. This limitation was less difficult to work with when considering only 60 proteins: the 30 higher and the 30 lowest MED scored proteins out of 553 *Mtb*'s predicted exported proteins (Figure 3).

### NetMHC version

The newest NetMHC software (version 3.2) offers the ability to predict epitopes for 57 MHC alleles (http://www.cbs.dtu.dk/services/NetMHC/), but there is not yet a stand-alone version available to download. The NetMHC version (3.0) used here is the previous version and offers the possibility to predict epitopes for 55 MHC alleles [34]. However, the changes in version 3.2, compared to version 3.0, include a small increment in the number of MHC alleles and the possibility to predict epitopes of lengths ranging from 8 to 14mers. The authors of version 3.2 advise that predictions of peptides longer than 11mers have not been extensively validated. They also advise caution regarding predictions involving 8mers, as some alleles might not bind 8mers to any significant extent (http://www.cbs.dtu.dk/services/NetMHC/). Moreover, most MHCs prefer peptides of 9mers and the alleles' set from the version 3.0 are still present in version 3.2 [18]. Therefore, epitope predictions based on version 3.0 are still valid to answer relevant biological queries.

### Are these pathogenic proteins?

The method presented here was initially conceived to predict antigenic proteins. Our approach is based on the fact that both antigenic and pathogenic proteins can be useful for vaccines and diagnosis and such targets can be revealed by the overall set of predicted epitopes and their concentrations in mature proteins. As related in the methods section, the *in silico *predicted exoproteins were ordered by decreasing MED score values. Following this sorting, the literature was searched for evidence proving or denying the contribution for the bacterial pathogenicity of each protein. The majority of the true positives presented here (Table 1) showed pathogenic instead of antigenic evidence (16 out 21 true positives), as detailed in the additional file 1. One protein (Rv3018c) has evidence for both antigenicity and pathogenicity simultaneously. In the same way, this criterion was also applied to the true negatives (Table 2), where seven out of 14 contrary cases fit into the pathogenic class instead of the antigenic one. Could these apparently unexpected results have a rationale? Could pathogenomics explain these findings? Pathogenomics is defined as the analysis, at genomic level, of the processes involved in bacterial pathogenesis caused by the interaction of pathogenic microbes and their hosts [35]. The identification of mutants showing altered pathology may be a useful framework to understand tuberculosis, but it is not clear how these phenotypes relate to the human disease [36]. Here, we presented evidence that *Mtb *pathogenic proteins have some of the highest MED scores within the *Mtb *genome.

## Conclusions

The search for new vaccine targets against prokaryotic microorganisms has been aided by extensive use of software motif recognition in sequences; nevertheless, considerable experimental effort is necessary to filter out the most promising candidates. The method presented here and the software available online can help to minimize experimental efforts by indicating promising prokaryotic proteins for target selection. The proposed method was called MED score and exhibits a strong relation to proteins proved to be important in the *M. tuberculosis *pathogenesis.

## Methods

### Genome data

The complete genome of *Mtb *H37Rv was obtained from the GenBank database under the NCBI identifier NC_000962. All coding sequences were selected and exported as amino acids in FASTA format using the annotation software ARTEMIS from the Sanger Institute.

### Prediction schema

**Figure 6:**
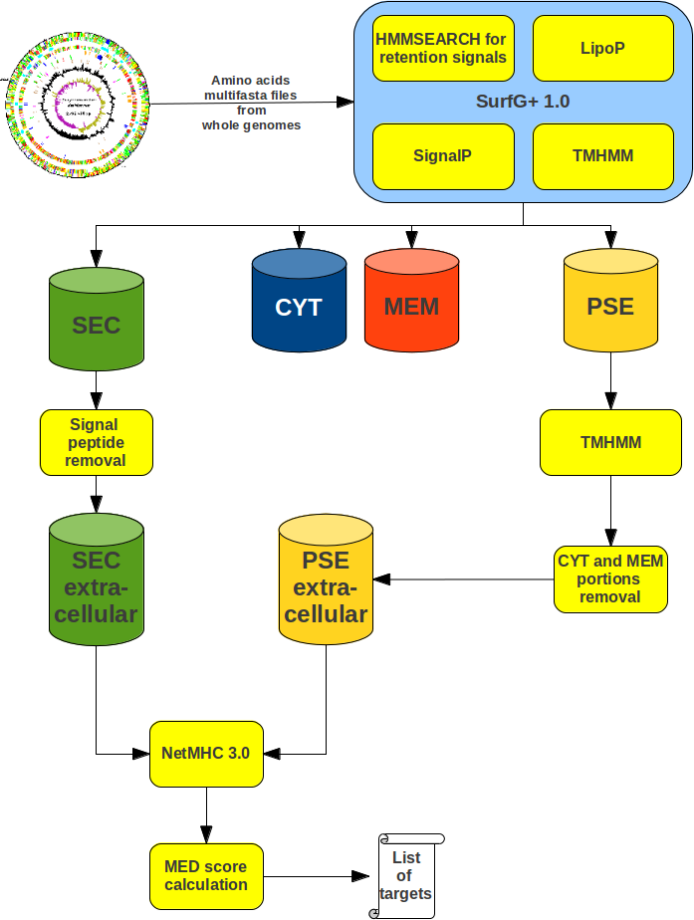
**Medpipe prediction schema**. Computational pipeline for MED score prediction.

Our software environment for MED predictions integrates SurfG+ [37], TMHMM [38] and NetMHC [18]. As seen in Figure 6, an amino acid MULTIFASTA file is first processed by SurfG+ to filter sequences predicted to be secreted (SEC) or potentially surface exposed (PSE). The SEC sequences have then their signal peptide intervals removed from the original sequence, maintaining only the predicted mature protein sequences for further processing. This step is also performed for PSE predicted sequences; however, another TMHMM prediction step is used on these sequences as SurfG+ does not store the TMHMM results concerning the mature portion of the sequences. These steps result in the creation of an artificial amino acid sequence from each original amino acid sequence predicted as SEC and PSE, containing only the concatenated original amino acid sequence portions that were predicted as the mature portions. The artificial amino acid sequences are then submitted to NetMHC, configured to predict all 55 possible MHC alleles within the software (version 3.0), and only the predicted strongly binding peptides are filtered for further processing. Finally, the MED score is calculated for each amino acid sequence according to Equation 1.

(1) $$\text{MED = }\frac{Predictions}{Chances}=\frac{Predicted\;epitopes*\left(\text{50}-\text{Average}\left(MHC\;Affnity\right)\right)}{Aminoacids\;length-Epitope\;length+\text{1}}$$

Equation 1 divides the number of linear predicted epitopes from each amino acid sequence by the number, for instance, of possible 9mers peptides overlapping windows. To ensure qualitative differentiation for this ratio calculation, the epitopes' MHC binding affinity average is also multiplied, after being normalized according to the maximum MHC strong binding affinity (50 nM). This calculation returns the Mature Epitope Density (MED), a number measured in nanomolar per mer (nM/mer) units. All amino acid sequences are ordered by descending MED score and presented as the final result. The prediction schema was implemented using a Linux shell script. The web server is hosted on Ubuntu OS, release 11.10 and the whole processing takes approximately 90 minutes for *Mtb *H37Rv amino acid sequences using a standard personal desktop computer.

### Control datasets

100 antigen and 100 non-antigen swissprot identifiers were obtained from a previous work [31]. These protein identifiers were retrieved from the Uniprot database [39], culminating in 107 and 121 amino acid sequences used as positive (Dplus) and negative (Dminus) control groups, respectively. To enrich our tests, a set of 38 *Mtb*'s proteins (Mtbplus) were similarly retrieved from the AntigenDB [40] and from Uniprot. The Mtbplus control group was obtained selecting the antigenic proteins from *M. tuberculosis *and filtering for those known as eliciting immune cellular responses.

### Evidence dataset

Sixty proteins out of the 553 *in silico *predicted as exported were chosen for detailed investigation of experimental proof concerning their capacity to induce cellular responses. In this regard, based on MED, 30 out of 60 proteins were designated as the lowest scored, and the other 30 were designated as the highest scored. An extensive literature search was carried out to look for evidence concerning whether these proteins were related to antigenicity or contribute to the bacterial pathogenicity. Supporting evidence for 39 out of 60 proteins was found, depending on whether a protein induces a cellular response, has evidence of frame shifts, has evidence of differential expression, is part of a known pathogenic protein family or has a cloning experiment that has failed. The complete evidence dataset and corresponding published evidence can be found in the additional file 1.

## Competing interests

The authors declare that they have no competing interests.

## Authors' contributions

ARS proposed the MED score concept, developed the prediction schema, collected literature evidence, wrote the paper and built the web tool. VBP and EB contributed to the manual curation of literature evidence and scientific discussions. MZT made substantial contributions to the design and interpretation of the manuscript. JB, JP and RR have given final approval of the version to be published and have given IT resources and expertise to maintain the web tool on line. AS and AM have given final approval of the version to be published. VA encouraged the research, BMC application, contributed with applied biological knowledge and has given final approval of the version to be published.

## Supplementary Material

Additional file 1 **A spreadsheet enumerating the complete list of supporting or contradicting evidence to the MED score hypothesis**. Click here for file

## Declarations

Publication for this article has been funded by CNPq - Conselho Nacional de Desenvolvimento Científico e Tecnológico - Brasil.

This article has been published as part of *BMC Genomics *Volume 14 Supplement 6, 2013: Proceedings of the International Conference of the Brazilian Association for Bioinformatics and Computational Biology (X-meeting 2012). The full contents of the supplement are available online at http://www.biomedcentral.com/bmcgenomics/supplements/14/S6.
